# Role of the Morphology and Polyphosphate in *Trichoderma harzianum* Related to Cadmium Removal

**DOI:** 10.3390/molecules16032486

**Published:** 2011-03-15

**Authors:** Adriana de Freitas Lima, Gabrielle Ferreira de Moura, Marcos Antonio Barbosa de Lima, Patrícia Mendes de Souza, Carlos Alberto Alves da Silva, Galba Maria de Campos Takaki, Aline Elesbão do Nascimento

**Affiliations:** 1 Master Course in Development of Environmental Processes, Catholic University of Pernambuco, Recife, PE, Brazil; 2 Nucleus of Research in Environmental Sciences, Catholic University of Pernambuco, 50.050-590 Recife, PE, Brazil; 3 Maurício de Nassau Faculty, Recife, PE, Brazil; 4 Biology Department, Federal Rural University of Pernambuco, Recife, PE, Brazil; 5 PNPD-CAPES, Catholic University of Pernambuco, Recife, PE, Brazil

**Keywords:** polyphosphate, *Trichoderma harzianum*, cadmium, morphology, ultrastructure

## Abstract

This study concerns the metabolism of polyphosphate in *Trichoderma harzianum*, a biocontrol agent with innate resistance against most chemicals used in agriculture, including metals, when grown in the presence of different concentrations of cadmium. The biomass production was affected by the concentration of metal used. Control cultures were able to accumulate polyphosphate under the conditions used. Moreover, the presence of cadmium induced a reduction in polyphosphate content related to the concentration used. The morphological/ultrastructural aspects were characterized by using optical and scanning electron microscopy, and were affected by the heavy metal presence and concentration. The efficiency of cadmium removal revealed the potential of the microorganism for use in remediation. The data indicate the potential for polyphosphate accumulation by the fungus, as well as its degradation related to tolerance/survival in the presence of cadmium ions.

## 1. Introduction

The physiological responses of microorganisms such as feasibility, metabolism, stages of growth and death are induced a wide range of processes of natural and/or anthropogenic order. Thus, as a result of environmental pressures, microorganisms quickly develop mechanisms of adaptation/ resistance to deal with adversity. Thus, researches related to the assessment of cellular responses are key to access the population heterogeneity, environmental quality, microbial ecology seeking for the understanding of the pollution by xenobiotics and for the relevance of their potential use in the process of detoxification/remediation [1,2,3,4].

The study of interactions between heavy metals and microorganisms has been focused on mechanisms of transformation and conversion of metal ions by the reduction in different polluted environments, in the selection and use of resistant organisms as indicators of toxicity for other life forms, as well as analysis of mechanisms, determinants and transference of resistance. Thus, trials related to biochemical and physiological behaviors are essential tools for the elucidation of these phenomena [5,6].

Cadmium is recognized for its toxicity throughout the food chain, and is one of the toxic components of industrial and municipal waste. The metal has no essential biological function and exhibits mutagenic, carcinogenic, phytotoxic and ecotoxic effects, being extremely toxic, due to its concentration, for all living systems, particularly for mammals, including humans. The toxic effects of the metal are a consequence of its ability to inactivate enzymes containing sulphydryl groups and may inhibit oxidative phosphorylation. Additionally, it can compete with other metals such as zinc and selenium for inclusion in metallo-enzymes and competes with calcium for the sites of connection with regulatory proteins such as calmodulin. These properties, together with its widespread use in batteries, make the cadmium one of the most common environmental pollutants [7,8,9,10,11,12].

The mechanisms of resistance of microorganisms in the presence of metals can be classified into two groups: metal dependant and metal independent [1,13,14,15,16]. One of the mechanisms is the sequestration of metal ions by polyphosphate [17,18]. In parallel, the existence of two mechanisms for the resistance associated to polyphosphate is proposed: reduction of intracellular concentration of the metal by the connection and/or hydrolysis of the polymer, which detoxify the metal [18,19,20,21].

The purpose of this study was to evaluate the behavior of *Trichoderma harzianum*, grown in the presence of different concentrations of cadmium, regarding the growth, morphology behavior, and polyphosphate profile and the ability of removal of the metal ion, considering the apparent relationship between polyphosphate and the increase of resistance/tolerance against heavy metals, which would indicate an application of polyphosphate-accumulator organisms for the bioremediation of areas contaminated with heavy metals.

## 2. Results and Discussion

### 2.1. Effects of cadmium in the growth, morphology and ultrastructure of Trichoderma harzianum

**Figure 1:**
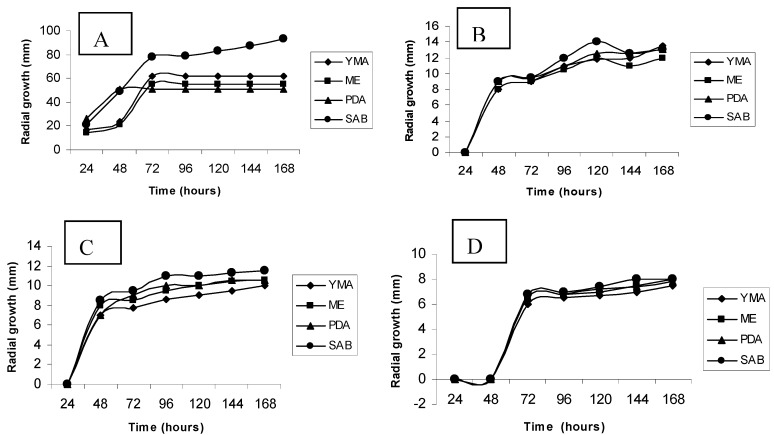
Radial growth of *Trichoderma harzianum* in different culture media, aiming to select a standard medium for the physiological and biochemical investigations. A- Control ; B- Treated with cadmium 1 mM; C- Treated with cadmium 2mM; D- Treated with cadmium 3mM. YMA (Yeast Malt Agar); ME (Malt Extract); PDA (Potato Dextrose Agar) and SAB (Sabouraud Dextrose Agar).

With the aim of selecting a standard medium for the physiological and biochemical experiments an initial study related to different growth media was performed. Figure 1A-D shows results for the radial growth obtained for the cultivation of *Trichoderma harzianum* in Yeast Mold Agar, Malt Extract, Sabouraud and Potato Dextrose Agar media, in absence and presence of cadmium, at concentrations of 1, 2 and 3 mM, respectively. 

From an analysis of graphics one can infer that the cultivation in the absence of metal in different media, results in different growth profiles, estimated by colony radial expansion. However, comparing the different media, higher radial growth was noted for cultivation in the Sabouraud medium. This study evaluated the percentage of relative growth of the organism for each test condition in relation to control. The results presented reveal the influence of cadmium on the radial growth of colonies of *Trichoderma harzianum*. The cultivation in different media resulted in variations of colony radial expansion, and revealed the inhibitory effect of the metal, which is directly related to its concentration. 

**Figure 2:**
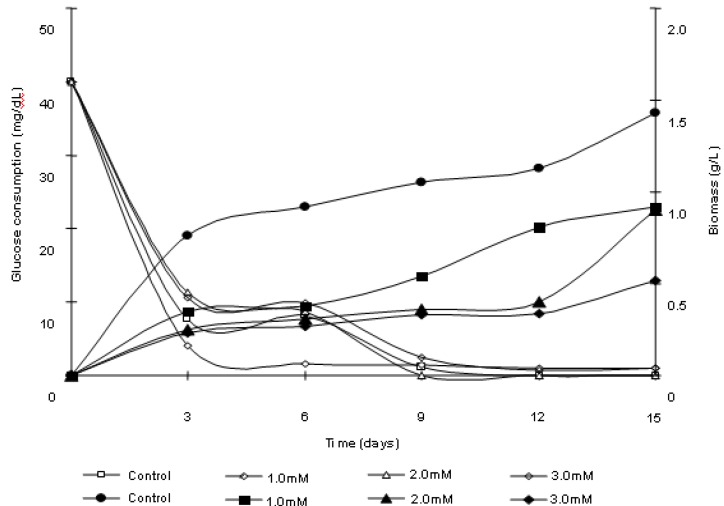
*Trichoderma harzianum* biomass production and glucose consumption in response to cadmium exposure.

Figure 2 shows the results of the biomass production of *Trichoderma harzianum* cultures in the presence and absence of cadmium. The presence of the metal induces a significant reduction of growth, as determined by the cellular biomass, in relation to control culture. A decrease in the biomass production proportional to the concentration of metal used was verified. It was observed that after 3 days a growth of biomass in control and treated samples occurred. Notwithstanding, treatment induced a reduction in biomass production, which amounted to 46.31%, 32.62% and 29.99% of the control culture production for concentrations of 1, 2 and 3 mM, respectively. At the end of the trial period for the culture treated with 1mM of cadmium, biomass obtained corresponded to 64.89% of control culture. For the treatments with 2 mM and 3 mM cadmium, the biomass corresponded to 63% and 36.83% of the mycelia mass obtained for control culture, respectively.

**Figure 3:**
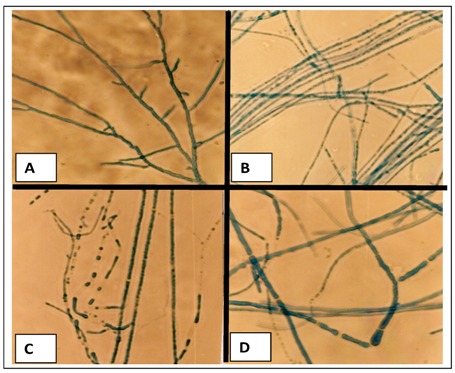
Morphological behavior of *Trichoderma harzianum*. A. Control culture; B. Culture treated with 1 mM of cadmium, C. Culture treated with 2 mM of cadmium and D. Culture treated with 3 mM of cadmium. 400×.

The behavior of the hyphae morphology, control and treated with cadmium, was evaluated through optical microscopy. The results are shown in Figure 3A-D. 

In control culture, the presence of chambered hyphae, with hyaline thin walls, is seen. Of note is the homogeneously stained and dense cytoplasm (Figure 3A). Furthermore, samples treated with cadmium showed smaller radial expansion, proportional to the concentration of metal in the culture medium. The cultivation in the presence of cadmium also prompted the emergence of intense septation and branching in hyphae. Samples submitted to the highest concentration of cadmium, showed the largest branch and septation of hyphae. 

**Figure 4:**
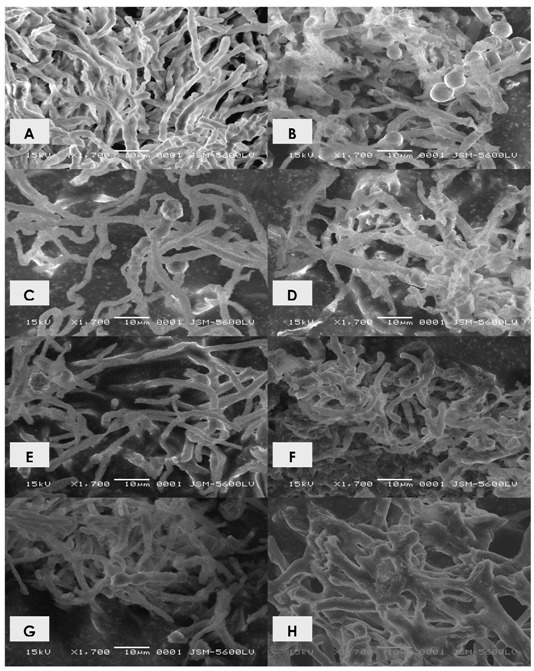
Electronmicrographs of *Trichoderma harzianum*. A. Control sample 3 days; B. Control sample 15 days; C. Sample grown in 1 mM of cadmium 3 days; D. Sample grown in 1 mM of cadmium in 15 days; E. Sample grown in 2 mM of cadmium 3 days; F. Sample grown in 2 mM of cadmium 15 days; G. Sample grown in 3 mM of cadmium 3 days; H. Sample grown in 3mM of cadmium in 15 days. 1.700×.

In parallel, it is possible to perceive that the cytoplasm of hyphae grown in the presence of cadmium seems to be heterogeneous and granular in appearance. The cultivation in medium containing the heavy metal induced a most intense cytoplasmic granulation (Figure 3B, 3C and 3D, 1 mM, 2 mM and 3 mM of cadmium, respectively). Through the ultrastructural study it was possible to verify the emergence of variations in the fine structure of the organism submitted to cultivation in the presence of cadmium. The results are shown in Figure 4A-F. 

For the control samples with 3-days of cultivation it was verified the presence of abundant and homogeneous mycelium, with elongated hyphae, chambered, in the form of sticks (Figure 4A). For samples with 15 days of cultivation, many reproductive structures were observed. The mycelium exhibited low electron density (Figure 4B). Moreover, samples treated with 1 mM of cadmium, 3 days showed more scarce mycelium, twisted, intense branching, increased electron density; shortened hyphae were also viewed (Figure 4C-D). Similar fine structure was observed in the samples treated with 2 mM of cadmium. However, higher electron density, patterns of branching and shortening of hyphae were observed for samples treated with 1 mM (Figure 4E-F). For samples grown in the presence of 3 mM, Figure 4G-H, the intensity of the changes mentioned were greater. Additionally, hyphae with 15 days of cultivation had become more twisted and thick that those treated with 1 mM and 2 mm of cadmium. An intense change in the branching pattern of hyphae was noted. The observed changes were directly related to the concentration of metal. 

### 2.2. Polyphosphate behavior of Trichoderma harzianum in the presence of cadmium

**Figure 5:**
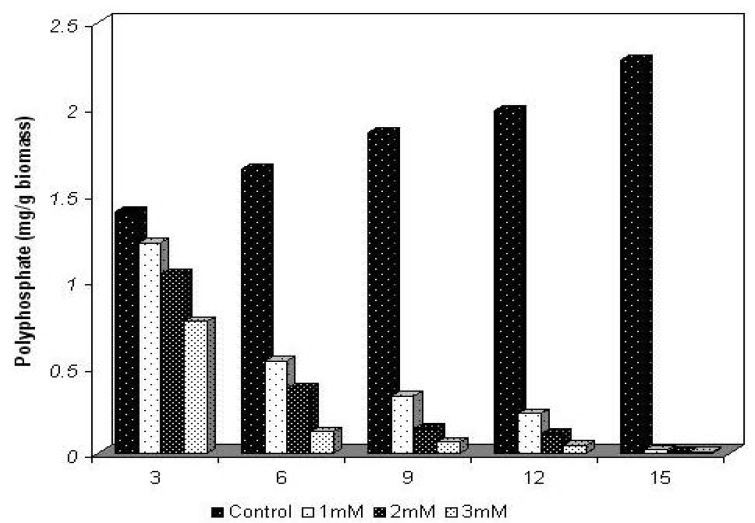
The physiological behavior of *Trichoderma harzianum* polyphosphate, cultivated in the absence and presence of cadmium.

The physiological behavior for the content of polyphosphate of *Trichoderma harzianum*, cultivated in the absence and presence of 1, 2 and 3 mM concentrations of cadmium, during intervals of 3, 6, 9, 12 and 15 days is shown in Figure 5.

For the control sample, in the absence of the metal, a progressive increase in the cellular polyphosphate content occurred. Values corresponding to 1.4 mg/g biomass, 1.65 mg/g biomass, 1.86 mg/g biomass, 1.987 mg/g biomass and 2.28 mg/g biomass were obtained during the experimental period. By the end of the experiment an increase of approximately 81% in the content of polyphosphate was reached, resulting in the ability of synthesis and accumulation of polyphosphate by the mycelium of isolate of *Trichoderma harzianum* tested. 

On the other hand, in cultures treated with 1 mM, 2 mM and 3 mM of cadmium, reductions of 13%, 25% and 45% respectively, in the content of polyphosphate, were observed within the first 3 days of cultivation in relation to control culture, although an increase in biomass production had occurred. At the end of the trial period the content of total cellular polyphosphate exhibited a reduction of approximately 99% for all cultures grown in the presence of the metal.

### 2.3. Removal of cadmium by Trichoderma harzianum

**Figure 6:**
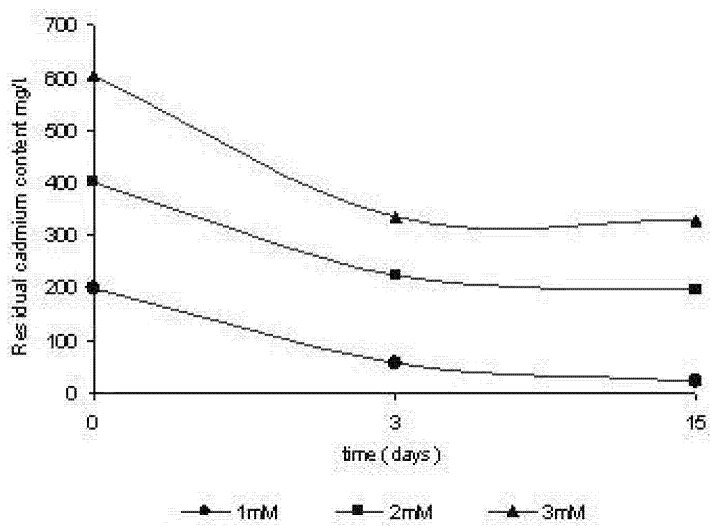
Removal of cadmium ions of culture by *Trichoderma harzianum*.

Tests were carried out in order to assess the change in the concentration of metal over cellular growth, through the determination of residual concentrations of cadmium in the cultive supernatant. The results are shown in Figure 6. It appears that there was a decrease of cadmium in the culture over time. Apart from that, the removal was dependent on the initial concentration used. The percentage of removal in the third day of cultivation accounted for 71.43%, 43.87% and 44.20% for 1 mM, 2 mM and 3 mM of cadmium, respectively. At the end of the trial period (15 days) percentages of removal of 87.13%, 50.57% and 45.69% were obtained for the cultures treated with 1 mM, 2 mM and 3 mM respectively. The data suggest that the removal process occurs at a higher speed during the beginning of cell growth, followed by a decrease in the process over fifteen days of cultivation.

### 2.4. Efficiency of removal of cadmium by Trichoderma harzianum

**Figure 7:**
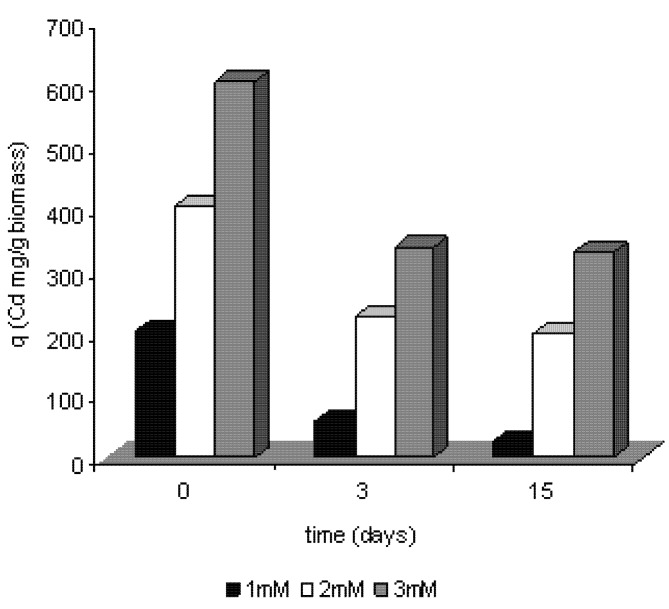
Efficiency of cadmium ions removal by the mycelium of *Trichoderma harzianum*.

Figure 7 shows the relationship between the amounts of metal biosorbed per unit of biomass. The efficiency of removal was carried out according to the equation q = (C_0_ - C_f_)/m. The data demonstrates that the efficiency of removing the metal of culture increases by the increase of the initial concentration of metal ion, resulting in 101.12 mg/g/biomass, 176.37 mg/g/biomass and 289.95 mg/g/biomass for concentrations of 1 mM, 2 mM and 3 mM, respectively. It is obvious a relation with the cell growth stages, resulting from a lower production of biomass over time of cultivation, and removal of cadmium. At the end of the trial period occurred a decrease in the efficiency of removal of cadmium by the total biomass, occurring a maximum removal of 289.95 mg/g/biomass for the concentration of 3 mM.

### 2.5. Discussion

The application of biomaterials for metal removal has attracted the attention of numerous researchers. Bacteria, fungi and algae, as well as products derived from such organisms, have the potential to remove many chemicals [1,4,16,22,23].

The removal of heavy metals or their stabilization is the first steps towards detoxification of contaminated environments. Thus, the remediation of environments contaminated with heavy metals is a complex problem and has attracted the attention of many researchers and industries. Conventional physical-chemical methods to remove heavy metals in several environments include chemical reduction, electro-chemical treatment, ion exchange, and precipitation and evaporation recovery. However, such processes exhibit significant disadvantages, such as incomplete removal, high energy and reagent consumptions, besides generation of other toxic products the high cost of recovering them [24,25,26].

Among the procedures used by microorganisms for metal detoxification can be cited: precipitation as phosphates, carbonates and sulfides; volatilization by methyl groups, physical exclusion by electronegative components in membranes and polymeric extracellular substances, efflux systems dependent on energy and intracellular sequestration by cysteine-rich protein of low molecular weight [1,2,4]. In general, the resistance includes a variety of strategies to deal with toxic concentrations of metals in the environment [7,27]; such strategies exist to prevent the entry of the metal into the cell or to actively pump out the metal from the cell [1,4,5].

Morphological changes as well as variations in cellular growth patterns of eukaryotes and prokaryotes are cited as effects induced by contact with cadmium. The intensity of responses is related to the time of contact and the metal concentration. Such changes are possibly related to changes in the structure/permeability of the cytoplasmic membrane, producing gradual alterations in morphology and loss of metabolic activity [6,9,15,16,27,28,29,30].

The metabolic and genetic diversity of fungi has been exploited for many years with the aim of obtaining industrial and biotechnological products in response to chemical and physical conditions of their environment [25,29]. Additionally, fungi have been used for the treatment of industrial and urban waste and residues. The potential of this usage lies in their molecular arsenal, and biomass produced in response to the environmental contamination [4,5,22].

The toxic effects of cadmium on microorganisms are well documented and derive from many mechanisms. For example, the binding of cadmium to sulphydryl groups leads to protein dysfunction. In parallel, the binding of cadmium to nucleotides leads to the collapse of the DNA molecule. Such effects undoubtedly result in a prolonged lag phase in cell growth, decrease in cell density or death [4,8,16,19,32,33].

A comparison between the concentrations used in this research, considered high for microorganisms, and the data reported by Kim *et al.*, [31], testing the growth of *Bacillus* in presence of different metals, shows that only an isolate was able to grow in 2 mM and 3 mM of lead, copper and zinc. Furthermore, the authors showed that concentrations between 0.9 mM and 1.9mM of cadmium completely inhibited the growth of the bacteria. In addition, the authors demonstrated a relationship between the decrease in total protein content and the presence of metals in the culture medium. 

Some reports point out the relationship between components of the culture medium, such as sources of carbon, nitrogen and phosphate, and their utilization processes during growth in the presence of heavy metals. Generally, the microorganisms evaluated exhibited higher reduction of biomass when cultivated in the presence of metal ions, even in low concentrations, occurring inhibition of growth and induction of metal accumulation [6,7,22,26].

On the other hand, some data show that metals such as cadmium are usually toxic for microorganisms, even at very low concentrations, which results in a reduction of the biomass production for different microorganisms [1,2,31].

Lopez and Vazquez [26] demonstrated that the presence of cadmium inhibited cell growth of *Trichoderma atroviride* in 50% to concentrations lower than 1 mM. Additionally, different variations of growth were observed in the presence of cadmium in cultures of *Rhodotorula* sp. Variations in the duration of the stages of cell growth and the production of biomass were viewed in different concentrations of the metal ion, usually lower than 1 mM [23,29]. In this study, however, it was possible to verify that the *Trichoderma harzianum* isolate evaluated showed high potential for tolerance/resistance in the presence of cadmium, in concentrations of 1 mM, 2 mM, and 3 mM. 

The literature shows that microorganisms with the ability to accumulate polyphosphate may be used in bioremediation of sewage contaminated with heavy metals, as several studies show the combination of polyphosphate granules with cations and heavy metals. In addition, the apparent relationship between polyphosphate and the increase of resistance or tolerance of some microorganisms to heavy metals strengthens its biotechnological potential in removal of these elements [17,18,20,27,34].

Some studies assert the action of polyphosphate in precipitation of metals, being the polymer a potential candidate in procedures for removal of polluting agents according to their ability of chelating for divalent metal [17,18,21,33,34].

It has been proposed that the cells use polyphosphate to detoxify only when the metal ions are internalized. However, recent evidence suggests that the polyphosphate is degraded during growth in the presence of metals [17,18,33]. The ability of synthesis and degradation of polyphosphate, determined in prokariots is important for the emerging phenomenon of tolerance to heavy metals, in contrast with the data that suggests that only a high intracellular polyphosphate content determines the phenomenon [17].

Alvarez and Jerez [35] support the model of detoxification of heavy metals, in which the metal ions stimulate the hydrolysis of polyphosphate, and the complex metals-polyphosphates are transported out of the cell possibly as a functional mechanism of microbial tolerance/resistance to the presence of these pollutants.

The decline in the content of polyphosphate in cells grown in the presence of cadmium indicates an increase in the cell energy demand, which would determine the possible role of the polymer as a reserve, being degraded during cell growth period.

The isolate of *Trichoderma harzianum* used in this study was able to accumulate polyphosphate over growth. In the presence of different concentrations of cadmium a significant degradation of polyphosphate occurred, which may indicate its use in remediation of water contaminated with heavy metals in sewage treatment plants in the process of removal of phosphate and heavy metals.

Data from literature on cadmium removal from culture media indicate that the process for most of the organisms tested, exhibits two stages: an initial one in which the sorption is characterized to be rapid and occurs in a short period of time, followed by a second stage, with a lower speed, and that contributes only a little to the total metal sorption [2,6,22]. Kappor and Viraraghavan [5] also reported that a higher rate of metal removal occurs in the early stages of growth. 

Wang *et al.* [29] demonstrated the ability of growth in concentrations of cadmium above 5 mM in a strain of *Pseudomonas aeruginosa*, isolated from deep marine waters. The organism was able to remove over 99% of cadmium of the means of cultivation, during the exponential growth phase.

Data gathered from this study corroborate those of literature in relation to the behaviour of removal in *Trichoderma harzianum*. The removal of metal ions by fungi can offer an alternative method for biological remediation. Fungi are applied in a large number of industrial processes, which can serve as a constant and economic source of biomass. Additionally, such organisms can easily grow through fermentation techniques of low cost [5]. Thus, researches on biosorption in fungi may represent an economic means for the treatment of areas contaminated with heavy metals.

Many species of fungi show potential for removal of cadmium. *Aspergillus terreus, Aspergillus flavus, Cladosporium cladosporioides, Fusarium oxysporum, Giocladium roseum, Penicillium* spp., *Mucor rouxii, Helminthosporium* sp, *Talaromyces helicus, Trichoderma koningii, Trichoderma harzianum*, and *Saccharomyces cerevisiae*, isolated from a polluted industrial area, were all efficient in the removal of the metal in aqueous solution. Thus, these data show the ability to remove the metal ion cell for cell biomass through mechanisms that do not depend on the active metabolism [5,28,32,36,37].

The values obtained for the efficiency of removal of cadmium vary depending on the concentrations of the metal used in the experiments. For example, Malik [22] reported the ability of isolates of *Giocladium roseum* to remove cadmium with a performance of 184 mg per gram of biomass produced. Moreover, Kapoor and Viraraghavan [5] published values ranging between 0.4 mg/g and 71 mg/g.

The results of this paper, compared with the literature, showed a promising capacity for removal of cadmium by the mycelium of *Trichoderma harzianum* during its growth in the presence of the metal, considering the high concentrations used.

Additionally, the tests also show the ability to remove the cadmium from the culture medium, meaning that the process depends on the active metabolism and cellular growth as a result of tolerance shown by the organism to the presence of the metal.

Although a reduction in the production of biomass has been found, indicating the effect of metal on cellular growth, total cadmium removed by the cells increased, being related to the concentration of metal used. Thus, this study shows for the first time that the isolate of *Trichoderma harzianum* used is capable of growing at high concentrations of cadmium, and consequently, the microorganism can potentially be used in bioremediation of systems contaminated with that metal.

## 3. Experimental

### 3.1. Microorganism and culture conditions

The isolate of *Trichoderma harzianum* UCP 1571 was obtained from the Culture Collection of the Núcleo de Pesquisas em Ciências Ambientais - Universidade Católica de Pernambuco - Brazil, included in the Rede Nordestina de Microrganismos do Norte e Nordeste (RENEBRA) and registered in the World Federation Culture Collection (WFCC). The isolate was maintained in Potato Dextrose Agar (PDA) at 5 °C. The fungus was cultivated in Sabouraud in order to produce spores and incubated at 28 °C for 6 days, for the production of pre-inoculum. 

### 3.2. Culture media screening by radial growth

An initial screening to select the appropriate medium for experimental procedures was performed by using the radial growth. Pre-inocula corresponding to cultures discs, with one centimeter in diameter, were inoculated in Petri dishes, containing different culture media: Malt Extract (ME), Potato Dextrose Agar (PDA), Sabouraud Dextrose Agar (SAB) and Yeast Malt Agar (YMA), containing cadmium chloride, prepared in distilled and deionized water, pH 6.0, calibrated with the use of sodium hydroxide 1N and acetic acid to 10%, at concentrations of 1 mM, 2 mM and 3 mM. The cultures were incubated at 28 °C for 7 days. Control-samples were grown according to the procedures cited without the metal. The growth was evaluated through the radial expansion, measured by the diameter of colonies, in millimeters, every twenty-four hours of incubation. The results are expressed as arithmetic average of triplicates. 

### 3.3. Growth curves

Erlenmeyer flasks of 300 mL of capacity, containing 150 mL of liquid Sabouraud were inoculated and incubated at 28 °C, in a orbital shaker (250 Hertz), for fifteen days. Samples were collected with 3, 6, 9, 12 and 15 days of cultivation and submitted to lyophilization for determination of biomass production by dry weight. Control samples were prepared, under the same conditions, without cadmium. All results were expressed in average of five replicates.

### 3.4. Effects of cadmium on the cell morphology

Samples of *Trichoderma harzianum* cells, grown under different experimental conditions, were collected with the help of a platinum loop, and deposited directly on the surface of glass layers, containing a drop of Aman Blue and covered by a laminule. The samples were observed under a Nikon Microscope model Alphaphot 2 Y52, and photographed with a Nikon camera FDX-35.

### 3.5. Ultrastructural analysis - scanning electronic microscopy

Samples collected within 15 days of cultivation, were washed twice in PBS, pH 7.2, for 10 minutes. Then they were fixed with 2.5% glutaraldehyde in 0.1 M phosphate buffer, pH 7.4, for 1 hour at room temperature. After the stage-setting, all samples were again washed twice with phosphate buffer, for 10 minutes. This procedure was followed by the post-fixing with osmium tetroxide 1% in phosphate buffer, for 1 hour at room temperature, in absence of light. Then the samples were once again washed with 0.1 M phosphate buffer, and submitted to the process of dehydration. The dehydration of the samples was done with ethanol, in concentrations of 50%, 70%, 90% (5 minutes for each exchange) until the proportion of 100% (three times, 10 minutes each exchange). After this step, the samples were submitted to the critical point, followed by the assembly in support of aluminum and subsequent gold metallization. Once prepared, samples were examined and photographed in the Scanning Electronic Microscope, JEOL LV 5600, operating at 20 KV. 

### 3.6. Extraction and dosage of total polyphosphate

The total intracellular polyphosphate was extracted and determined according to the method described by Mcgrath and Quinn [38]. Samples of mycelium (7 mg dry weight) of the fungal control and treated cultures, harvested at 3, 6, 9, 12 and 15 days of culture were collected, processed and washed twice in a solution of NaCl 1.5 M EDTA containing 0.01 M and NaF 1mM. The cell pellet was resuspended in wash buffer (1.5 mL) and sonicated on ice, for 24 minutes period with 2 minute intervals at 16 KHz. The resulting homogenate was centrifuged at 12.000 g for 5 minutes to remove cell debris. To determine total intracellular polyP, concentrated HCl (100 µL) was added to cell extract (0.5 mL) and heated at 100 °C for 45 minutes. The phosphate released was determined by the colorimetric method (Fiske-Subbarow [39], based on the reaction of inorganic phosphorus with ammonium molybdate in acid solution, measured spectrophotometrically at 600 nm, and whose intensity is directly proportional to the phosphorus concentration. A pattern curve was designed, using a solution of phosphorus (0.5–5.0 mg/dL). The readings were carried out in Spectronic model GENESYS 2 digital spectrophotometer. The polyphosphate concentration was expressed in milligrams per deciliter of phosphorus and given as average of triplicates. A non-hydrolyzed sample was used as a control to determine the level of phosphate free into the cell. The total polyphosphate is expressed in milligrams per gram of biomass.

### 3.7. Kinetics of cadmium removal

To provide data for the biotechnological treatments based on the understanding of the removal process the amount of metallic ion removed by g of the biomass (q) and the efficiency of removal were calculated. Thus, to assess the kinetics of cadmium removal by the mycelium of *Trichoderma harzianum*, samples from supernatants of cultures collected in the intervals of 3 and 15 days were used. The concentration of metal was determined by plasma spectroscopy, with specific lamp for cadmium. A pattern curve was designed. Experiments were conducted in triplicate and the averages were used in the data. The efficiency of the metal removal - q (mg of metal ion/gram of biomass) was calculated using the following equation:

q = (C_0_ − C_f_)/m

where C_0_ and C_f_ corresponded to the initial and final concentrations of the metal (mg/L), respectively, and m is the biomass on dry weight (g) [32].

## 4. Conclusions

In this study, a strain of *Trichoderma harzianum* grown in the presence of cadmium, showed growth ability in high concentrations of cadmium, being, however, the growth profile related to the concentration of the metal ion. Besides, it was shown that the organism in control cultures showed potential for accumulation of polyphosphate, and that such behavior can be applied for the removal of metals in sewage. Furthermore, the polyphosphate profile was amended as a result of the presence of different concentrations of cadmium, demonstrating that the polymer is degraded. The ability to accumulate and hydrolyse the polymer can be very important, not only for the cell survival in a contaminated environment, but also in its application in the bioremediation processes. Additionally, the results for the efficiency of removing the metal of the means of cultivation, over cell growth, point to the potential of the isolated for studies of remediation of metals.
